# Red cell index: A novel biomarker for 3‐month mortality in acute ischemic stroke patients treated with intravenous thrombolysis

**DOI:** 10.1002/brb3.2170

**Published:** 2021-05-04

**Authors:** Meizi Qian, Xinbo Zhou, Beibei Gao, Honghao Huang, Chenguang Yang, Tian Zeng, Jiamin Shen, Jingyu Hu, Fangyue Sun, Shengqi Li, Xuerong Huang, Guangyong Chen

**Affiliations:** ^1^ Department of Anesthesia The First Affiliated Hospital of Wenzhou Medical University Wenzhou China; ^2^ Department of Neurology The Third Affiliated Hospital of Wenzhou Medical University Wenzhou China; ^3^ School of the First Clinical Medical Sciences Wenzhou Medical University Wenzhou China; ^4^ Department of Internal Medicine The Third Affiliated Hospital of Wenzhou Medical University Wenzhou China

**Keywords:** biomarker, mortality, prognosis, red cell index, stroke

## Abstract

**Background:**

The red cell index (RCI) was described as a biomarker for evaluating respiratory function in previous studies, but the relationship between RCI and stroke, remained a mystery. The present study aimed to probe the association between RCI at 24‐hr and 3‐month mortality and functional outcomes among acute ischemic stroke (AIS) patients treated with recombinant tissue plasminogen activator (r‐tPA).

**Methods:**

A total of 217 AIS patients between January 2016 and January 2019 were recruited in this retrospective study. AIS patients were grouped in terms of RCI tertiles. Predictive factors were confirmed via multivariate logistic regression analysis. The receiver operating characteristic (ROC) was used to assess the ability of RCI in predicting mortality. In addition, the risk of 3‐month all‐cause mortality was evaluated by Cox proportional hazard model.

**Results:**

We grouped AIS patients into tertiles with the purpose of comparing clinical factors and RCI levels. Multivariate logistic regression analysis presented that RCI (odds ratio [OR] = 1.443, 95% confidence interval [CI] [1.167–1.786], *p* = 0.001) was an independent biomarker for 3‐month all‐cause mortality. The best cutoff value of RCI was 2.41 (area under the curve [AUC] = 0.639, 95% CI [0.501–0.778], *p* = .032), with a sensitivity of 40.9% and a specificity of 89.7%. Cox survival analysis demonstrated a positive significant correlation between RCI (hazard ratio [HR] = 1.332, 95% CI [1.148–1.545], *p* < .001) and mortality risk.

**Conclusion:**

RCI, a potential predictor, was significantly associated with 3‐month mortality in AIS patients with r‐tPA.

## Graphical abstract

This is the first article on the prognostic value of red cell index in AIS patients treated with r‐tPA. There is no correlation between red cell index and 3‐month poor outcome. Red cell index is significantly associated with 3‐month mortality.

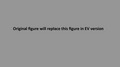

## 1 INTRODUCTION

Stroke is the leading cause of death and disability among adults worldwide, imposing enormous economic and social burdens on patients’ families. Acute ischemic stroke (AIS) is the most common type of stroke in China (Liu et al., 2011). Current treatment strategies for AIS include intravenous recombinant tissue plasminogen activator (r‐tPA) and endovascular mechanical therapy (Altintas et al., 2016). In AIS managements, despite that endovascular thrombectomy can be performable to people across a wide span of age and initial stroke severity, thrombolysis with r‐tPA is recognized and gradually popularized these years (Goyal et al., 2016). Nevertheless, after recanalization, several complications such as intracerebral hemorrhage and cerebral edema may arise, affecting the function outcomes (Cheripelli et al., 2016; Leng & Xiong, 2019).

Hypoxia, which may have adverse effects on cells in the ischemic penumbra, usually broke out during the first few days after the onset of AIS and tended to cause secondary brain damage (Roffe et al., 2003). In the clinical treatment, supplementation oxygen which was aimed to increase blood oxygen can constantly ameliorate prognosis, but sometimes it may do harm to patients (O'Driscoll, 2017). Hemoglobin, a special protein in red blood cells that transports oxygen, was considered to be of prognostic value for anemia at the lower level and for vascular blood clotting at the higher level. Previous studies had shown that cardiovascular system could be influenced by hemoglobin concentrations via blood viscosity, oxygen supply, and vasoconstriction (Cabrales et al., 2011). Elevated hemoglobin levels were associated with the risk of hypertension, blood pressure, and carotid atherosclerosis, while low ones were related to the risk of cardiovascular death and major bleeds, which could probably enlarge infarct size and accelerate infarct growth (Atsma et al., 2012; Kalra et al., 2017). There were relationships between hemoglobin treated with mechanical thrombectomy or arterial thrombolysis and prognosis (Ling et al., 2020; Sun et al., 2020). Moreover, a recent article had demonstrated that elevated hemoglobin levels in acute phase were associated with poor outcomes at 3 months after ischemic stroke, which further illustrated the association between the three: hemoglobin, prognosis, and mortality (Guo et al., 2019).

Inflammation and the immune response that were also key elements in stroke played a significant role in the pathogenesis of ischemic stroke and its subtypes, leading to infarct progression through reperfusion injury though under the treatment of thrombolysis or thrombectomy. The immune system was involved in the brain damage caused by ischemia, while the damaged brain played an immunosuppressive role, contributing to deadly infections. The inflammation system was involved in all phases of the ischemic cascade, from early injury events to late regeneration (Iadecola & Anrather, 2011). Lymphocyte counts provided insight into the inflammatory process and reflected the effect of acute physiological stress. Lower lymphocyte counts were significantly associated with the poor functional outcome with increased probability (Kim et al., 2012). Relative decreased lymphocyte may mirror cortico‐related stress responses and sympathetic tone, resulting in the increasing production of proinflammatory cytokines and eventually increased the severity of ischemic injury (Acanfora et al., 2001). The activation and aggregation of platelet, a well‐known hemostatic functional hemocyte variable, not only played an intermediary role in thrombus formation, but also participated in the inflammatory process (Hvas, 2016), giving rise to the instability of plaque. Accumulated evidence indicated that a low platelet level was associated with the poor outcome of AIS patients (Mayda‐Domac et al., 2010). As for erythrocytes, during ischemic stroke, red blood cells went through oxidative and hydrolytic changes, also causing inflammatory processes and changes of cellular rheology. The latest articles manifested that alterations in the RBC membrane and dense matted deposits (DMDs) that caused blood cells to be trapped in the mesh played an essential part in the presence of thrombi, which had ulteriorly impacts on ischemic stroke (Pretorius & Lipinski, 2013).

Currently, a new indicator, the red cell index (RCI), has been put forward, taking hemoglobin (Hb), lymphocyte (Lym), platelet (Plt), and red blood cell (RBC) into regard, and was calculated using the following equation: (RBC ×Hb) / (Lym ×Plt) (Guang et al., 2018). Theoretically, the RCI level was inversely proportional to the respiratory function. Meanwhile, RCI could be deemed as a simple and resultful biomarker for evaluating respiratory function (Guang et al., 2018), but no studies mentioned the relationship between RCI and prognosis of ischemic stroke. To our knowledge, the prognostic value of RCI in AIS patients with r‐tPA infusion was discussed herein for the first time.

The present study aimed to evaluate the correlation between RCI at 24‐hr and 3‐month mortality, and functional outcomes among AIS patients treated with r‐tPA.

## 2 MATERIALS AND METHODS

### 2.1 Study population

**FIGURE 1:**
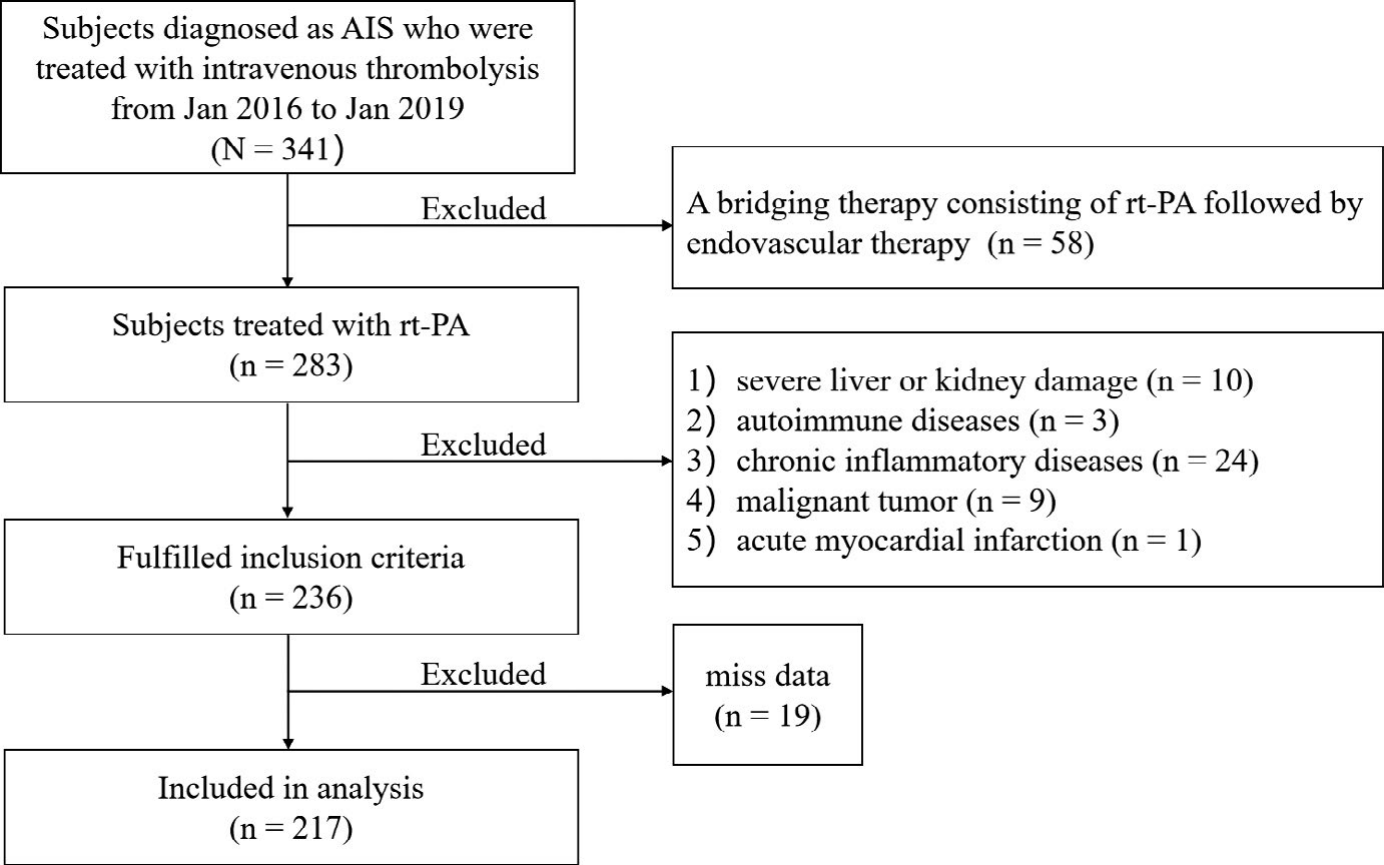
Flow diagram showing the patient selection process

As demonstrated in Figure 1, this retrospective study was based on a database of 341 patients with AIS treated consecutively with intravenous thrombolysis in the Third Affiliated Hospital of Wenzhou Medical University from January 2016 to January 2019.

Exclusion criteria were as follows: (1) a bridging therapy consisting of intravenous r‐tPA followed by endovascular therapy; (2) a therapy of urokinase thrombolysis; (3) severe liver or kidney dysfunction; (4) autoimmune diseases; (5) chronic inflammatory diseases or malignant tumor; (6) acute myocardial infarction; and (7) incomplete data. Finally, 217 patients were included in the current study.

The study was approved by the Ethics Committee of the Third Affiliated Hospital of Wenzhou Medical University and was carried out in accordance with the Declaration of Helsinki. The Ethics Committee number for the study is YJ2020034. All patients or their relatives gave informed consent in writing.

### 2.2 Data collection

Demographic characteristics (age and sex), baseline vital signs (systolic blood pressure and diastolic blood pressure), baseline stroke risk factors (hypertension, diabetes mellitus, hyperlipemia, history of stroke and smoking, atrial fibrillation, and coronary heart disease) and the National Institutes of Health Stroke Scale (NIHSS) on admission and at 24 hr were reviewed from the database. In addition, we recorded 3‐month modified Rankin Scale (mRS) after onset of AIS via two trained physicians on phone interview.

### 2.3 Assessment of RCI

Blood samples were collected on 24‐hr admission and the complete blood count parameters, including RBC count (10^12^/L), hemoglobin level (g/L), lymphocyte count (10^9^/L), platelet count (10^9^/L) were measured. The RCI was estimated using the following equation: RCI = (RBC × Hb) / (Lym × Plt). In accordance with RCI at 24 hr, all patients were divided into tertiles.

### 2.4 Study endpoints

3‐month all‐cause mortality was the primary endpoint. The secondary endpoint was the functional outcomes evaluated by the mRS: a favorable outcome was defined as mRS ≤2, while an unfavorable outcome was defined as mRS >2. The severity of AIS was defined according to NIHSS scores, and thus, AIS‐population was grouped as follows: mild or moderate stroke (NIHSS scores: 0–10) and severe stroke (NIHSS scores: > 10) (Asberg et al., 2018).

### 2.5 Statistical analysis

Statistical analyses were processed using SPSS 25.0 software (SPSS Inc., Chicago, IL, USA). Distribution normality was tested by the Kolmogorov–Smirnov test. Continuous variables with a normal distribution were exhibited by mean ±standard deviation (*SD*) and analyzed by one‐way analysis of variance, while continuous variables that followed a non‐normal distribution were expressed as median (interquartile range [IQR]) and analyzed by Kruskal–Wallis test. Categorical variables are represented as percentage numbers and analyzed by chi‐square test or Fisher's exact test. Variables, which differed significantly with *p* values of <.05 in univariate logistic regression analysis, were acknowledged as covariates for multivariate logistic regression analysis. The area under the receiver operating characteristic curve (ROC) was used to evaluate the ability of RCI in predicting mortality and calculate the cutoff point. According to the cutoff point, the RCI level was dichotomized at high or low values while involved in the survival analysis. Cox multivariate proportional hazards regression analysis was used for assessing the risk of a future clinical event in an individual patient. A *p* <.05 was considered significant for all analyses.

## 3 RESULT

### 3.1 Characteristics of study subjects

The baseline demographic and clinical characteristics of eligible patients were summarized in Table 1. The study population comprised 136 males and 81 females with an average of 70 years (IQR, 60–78 years). According to RCI values at 24 hr, patients were divided into three groups (RCI <1.61; 1.61 ≤ RCI ≤2.55; RCI >2.55). RCI <1.61 was considered as the lower group, 1.61 ≤ RCI ≤2.55 was considered as the middle group, and RCI >2.55 was considered as the higher group. Among subgroups, no significant correlation was found in terms of age, RBC, Hb, SBP, DBP, history of hypertension, diabetes mellitus, stroke, and coronary heart disease. The number of males, NIHSS score on admission and at 24 hr, and percentage of atrial fibrillation and cardiogenic were higher in patients who had higher RCI values (*p* <.05). Lymphocyte, platelet and percentage of hyperlipemia, atherosclerosis, and small vessel occlusion were significantly lower than patients with lower RCI values.

**TABLE 1:** Characteristics of the study population

	Total (*n* = 217)	24 hr RCI			*p*‐value
		RCI lower than 1.61	1.61 ≤ RCI ≤2.55	RCI higher than 2.55	
		(72, 33.2%)	(72, 33.2%)	(73, 33.6%)	
Demographic data					
Age, years	70 (60–78)	68 (58–75)	69 (60–77)	73 (61–80)	.628
Male, *n* (%)	136 (62.67)	36 (50.00)	45 (62.50)	55 (75.34)	.007
Stroke risk factors, *n* (%)					
Hypertension	137 (63.13)	53 (73.61)	43 (59.72)	41 (56.16)	.071
Diabetes mellitus	41 (18.89)	19 (26.39)	11 (15.28)	11 (15.07)	.139
Hyperlipemia	28 (12.90)	16 (22.22)	8 (11.11)	4 (5.48)	.009
AF	68 (31.34)	13 (18.06)	26 (36.11)	29 (39.73)	.011
History of stroke	15 (6.91)	6 (8.33)	5 (6.94)	4 (5.48)	.795
CHD	15 (6.91)	3 (4.17)	4 (5.56)	8 (10.96)	.234
History of smoking	79 (36.41)	27 (37.50)	29 (40.28)	23 (31.51)	.533
Vital signs					
SBP, mmHg	159 (140–175)	159 (139–179)	158 (141–177)	161 (141–174)	.635
DBP, mmHg	88 (78–99)	87 (78–99)	90 (80–100)	88 (75–98)	.576
TOAST subtype, *n* (%)					
Cardiogenic	84 (38.71)	13 (18.06)	33 (45.83)	38 (52.05)	<.001
Atherosclerosis	85 (39.17)	40 (55.56)	23 (31.94)	22 (30.14)	
Small vessel	21 (9.68)	11 (15.28)	4 (5.56)	6 (8.22)	
Others or undetermined	27 (12.44)	8 (11.11)	12 (16.67)	7 (9.59)	
Stroke evaluation					
Baseline NIHSS score	8 (5–12)	7 (5–11)	7 (5–10)	10 (5–14)	.013
24 hr NIHSS	5 (2–11)	4 (2–9)	4 (2–8)	8 (3–16)	.001
Functional outcomes					
Unfavorable outcome	79 (36.41)	24 (33.33)	18 (25.00)	37 (50.68)	.005
Laboratory data					
RBC, 10^12^/L	4.36 ± 0.51	4.24 ± 0.52	4.41 ± 0.51	4.43 ± 0.47	.100
Hb, g/L	131.27 ± 15.25	128.72 ± 15.74	132.45 ± 15.70	132.86 ± 14.05	.242
Lymphocyte, 10^9^/L	1.50 (1.10–1.90)	2.00 (1.70–2.38)	1.50 (1.30–1.80)	1.00 (0.80–1.30)	<.001
Platelet, 10^9^/L	194 (155–230)	246 (209–281)	191 (166–215)	152 (132–180)	<.001

Abbreviations: AF, atrial fibrillation; CHD, coronary heart disease; DBP, diastolic blood pressure; Hb, hemoglobin; NIHSS, National Institutes of Health Stroke Scale; RBC, red blood cell; RCI, red cell indexSBP, systolic blood pressure.

### 3.2 Association between RCI values and clinical prognosis

**FIGURE 2:**
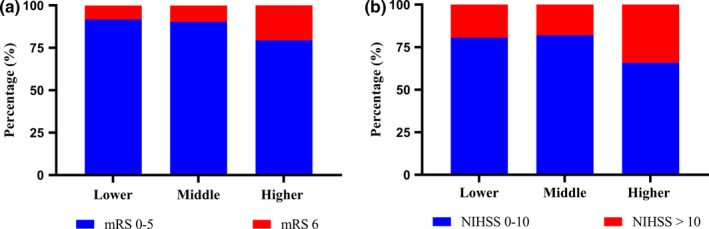
Primary outcomes according to RCI levels. (a) Distribution of modified Rankin Scale (mRS) scores at 3 months in patients treated with thrombolysis. (b) Distribution of National Institutes of Health Stroke Scale (NIHSS) at 24 hr in patients treated with thrombolysis

The distribution of mRS scores at 3 months and NIHSS at 24 hr in patients treated with thrombolysis were presented in Figure 2. Patients with higher RCI level tended to have higher stroke severity and poor prognosis at 3 months significantly. To research the factors that could predict the outcome of poor function, univariate and multivariate logistic regression analysis were conducted (Table 2). In univariate logistic regression, analysis showed that age, atrial fibrillation, percentage of cardiogenic, atherosclerosis, small vessel occlusion, history of smoking, RCI, and NIHSS before thrombolysis were discovered to be statistically correlated with poor outcome (*p* < .05). After adjusting for confounders, RCI did not show an autocephaly predictive factor of the poor outcome in AIS patients (odds ratio [OR] = 1.150, 95% confidence interval [CI] [0.975–1.358], *p* = .097).

**TABLE 2:** The logistic regression analyses of predictors to unfavorable functional outcome in 3 months

	Univariate Analysis			Multivariate Analysis		
	OR	95% CI	*p*‐value	OR	95% CI	*p*‐value
Age, years	1.048	1.022–1.075	<.001	1.030	0.998–1.063	.062
Sex	0.743	0.421–1.312	.306			
Hypertension	1.713	0.948–3.097	.075			
Diabetes mellitus	0.582	0.274–1.238	.160			
Hyperlipemia	1.908	0.858–4.243	.113			
AF	2.111	1.171–3.805	.013	1.108	0.432–2.845	.831
History of stroke	0.865	0.285–2.627	.798			
CHD	1.178	0.403–3.442	.764			
History of smoking	0.372	0.200–0.695	.002	0.557	0.260–1.194	.133
SBP, mmHg	1.010	0.999–1.021	.064			
DBP, mmHg	1.000	0.982–1.018	.970			
RCI	1.199	1.038–1.385	.014	1.150	0.975–1.358	.097
TOAST						
TOAST (cardiogenic)	1.000		.002	1.000		
TOAST (atherosclerosis)	0.397	0.211–0.748	.004			
TOAST (small vessel)	0.048	0.006–0.372	.004			
TOAST (Others or undetermined)						
NIHSS before thrombolysis	1.268	1.180–1.362	<.001	1.242	1.151–1.340	<.001

Abbreviations: AF, atrial fibrillation; CHD, coronary heart disease; DBP, diastolic blood pressure; NIHSS, National Institutes of Health Stroke Scale; RCI, red cell indexSBP, systolic blood pressure.

### 3.3 Association between RCI values and mortality

The primary end point (death) was observed in 20 patients. As can be seen from the univariate analysis, age (*p* = .002), sex (*p* = .031), history of smoking (*p* = .029), percentage of cardiogenic (*p* = .003), and atherosclerosis (*p* = .001), RCI (*p* = .001), NIHSS before thrombolysis (*p* < .001) were remarkably different. Multivariate analysis illustrated that sex (OR = 0.160, 95% CI [0.034–0.755], *p* = .021), NIHSS before thrombolysis (OR = 1.241, 95% CI [1.111–1.385], *p* < .001) remained crucial predictors of mortality. Beyond that, RCI (OR = 1.443, 95% CI [1.167–1.786], *p* = .001) was independently associated with the mortality. Higher RCI values represented the higher risk of all‐cause mortality. The result of logistic regression analysis was set out in Table 3.

**TABLE 3:** The logistic regression analyses of predictors to mortality in 3 months

	Univariate Analysis			Multivariate Analysis		
	OR	95% CI	*p*‐value	OR	95% CI	*p*‐value
Age, years	1.078	1.027–1.130	.002	1.053	0.994–1.116	.081
Sex	0.371	0.151–0.911	.031	0.160	0.034–0.755	.021
Hypertension	1.282	0.499–3.292	.605			
Diabetes mellitus	0.652	0.184–2.319	.509			
Hyperlipemia	0.650	0.143–2.945	.576			
AF	2.421	0.993–5.901	.052			
History of stroke	1.400	0.295–6.653	.672			
CHD	1.400	0.295–6.653	.672			
History of smoking	0.247	0.071–0.864	.029	2.061	0.334–12.728	.436
SBP, mmHg	1.005	0.988–1.022	.553			
DBP, mmHg	0.991	0.963–1.020	.546			
RCI	1.307	1.122–1.524	.001	1.443	1.167–1.786	.001
TOAST						
TOAST (cardiogenic)	1.000		.003		1.000	
TOAST (atherosclerosis)	0.088	0.020–0.394	.001			
TOAST (small vessel)						
TOAST (Others or undetermined)						
NIHSS before thrombolysis	1.244	1.148–1.349	<.001	1.241	1.111–1.385	<.001

Abbreviations: AF, atrial fibrillation; CHD, coronary heart disease; DBP, diastolic Blood Pressure; NIHSS, National Institutes of Health Stroke Scale; RCI, red cell indexSBP, systolic blood pressure.

**FIGURE 3:**
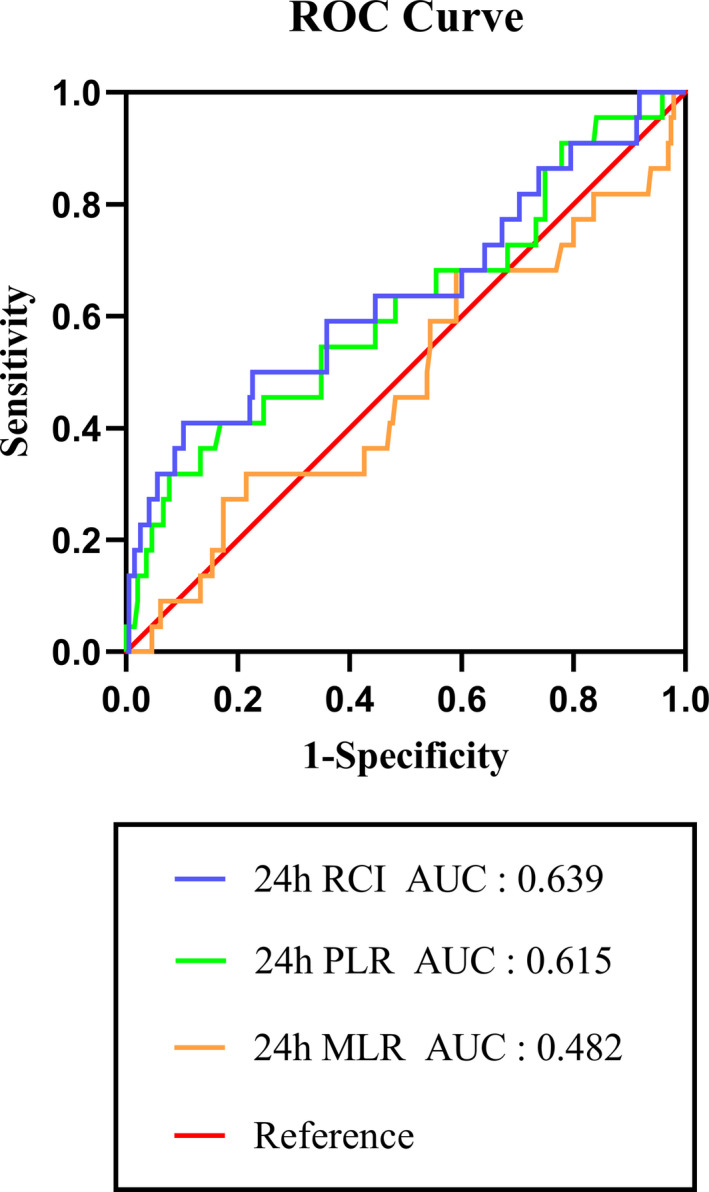
Receiver operating characteristic curve (ROC) of RCI, PLR, and MLR on the prognosis of AIS patients in mortality

ROC curve was performed to evaluate the ability of RCI, platelet to lymphocyte ratio (PLR), and monocyte to lymphocyte ratio (MLR) (Liu et al., 2020; Xu et al., 2019) in predicting the 3‐month mortality in AIS patients (Figure 3). The area under the curve [AUC] of RCI suggested higher degree of accuracy compared with the other two models, which illustrated that RCI had better predictive ability. Based on this analysis, the best cutoff value of RCI levels was found to be 2.41 (AUC = 0.639, 95% CI [0.501–0.778]), with a sensitivity of 40.9% and a specificity of 89.7%. And as suggested, RCI value was significantly correlated with mortality (*p* = .032).

**FIGURE 4:**
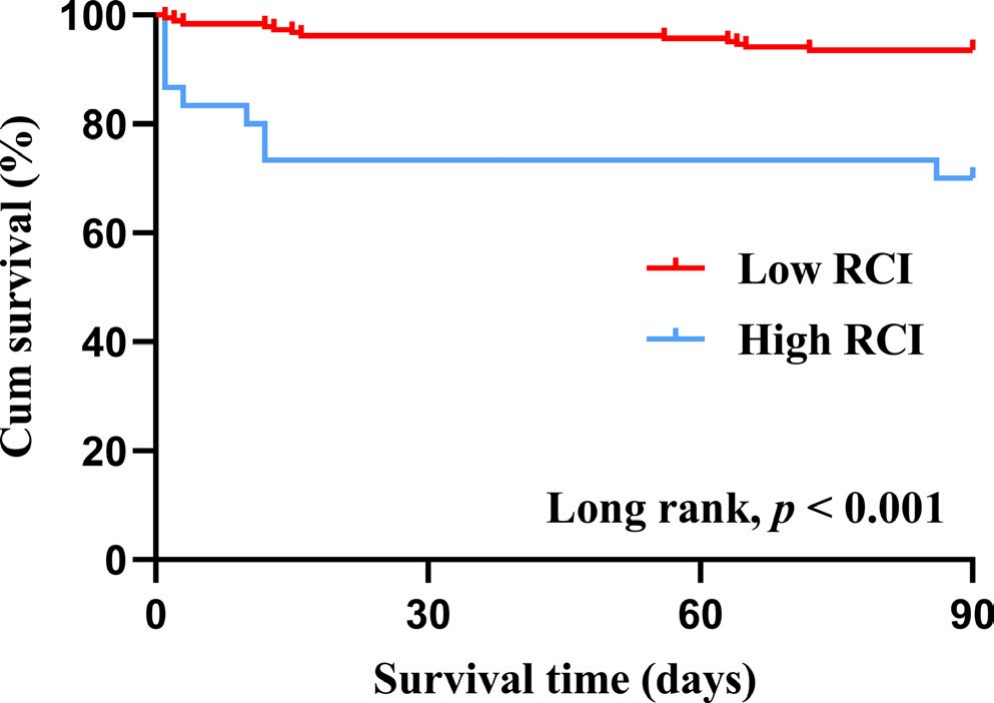
Kaplan–Meier curves comparing death rate of the 2 groups over 3‐month follow‐up. RCI, red cell index

In accordance with cutoff point of ROC curve, the RCI value was classified into high and low groups and survival curve was carried out (Figure 4) (Long rank, *p* <.001). Meanwhile, multivariate cox regression proportional hazard model analyses were further performed after adjusting the feasible confounding effect, showing a significant association between RCI (hazard ratio [HR] = 1.332, 95% CI [1.148–1.545], *p* <.001) and mortality risk (Table 4).

**TABLE 4:** The cox regression model analyses assessment the death risk

	Univariate Cox Regression Model			Multivariate Cox Regression Model		
	HR	95% CI	*p*‐value	HR	95% CI	*p*‐value
Gender	0.395	0.169–0.925	.032	0.267	0.081–0.876	.029
NIHSS before thrombolysis	1.198	1.134–1.266	<.001	1.170	1.091–1.254	<.001
RCI	1.262	1.132–1.406	<.001	1.332	1.148–1.545	<.001

Abbreviations: NIHSS, National Institutes of Health Stroke Scale; RCI, red cell index.

## 4 DISCUSSION

RCI was calculated using the equation: (RBC ×Hb) / (Lym ×Plt), taking four key indicators: RBC, Hb, Lym, and PLT. In this retrospective study, AIS patients with lower RCI values, treated with r‐tPA thrombolysis, had higher lymphocyte and platelet counts. And our data presented that RCI was significantly associated with 3‐month mortality. ROC curve further confirmed RCI level was an effective biomarker to predict 3‐month mortality. In addition, there was a positive correlation between RCI level and mortality risk, even after adjusting the potential confounding effect.

Hemoglobin, a well‐established marker to detect anemia, had been suggested to have possible pathophysiological pathways. The damage of endothelial cell could upgrade growth factor level, which strengthened hematopoiesis level (Wisniewski et al., 2020). And elevated blood pressure was prevalent in the acute period of ischemic stroke (Qureshi et al., 2007), activating the renin–angiotensin–aldosterone system and in turn causing the production of angiotensin‐2, vasoconstriction, and erythropoietin. In addition to the elevation of blood pressure, endothelial cell can also raise the hemoglobin level. Besides, haemorheology played a crucial role in the brain microcirculation, particularly in acute phase of ischemic stroke. A slightly decrease in blood flow may have influenced on cerebral function greatly. Elevated hemoglobin could not only increase blood viscosity, which affected coronary and cerebral blood flow, but also accelerate the aggregation of red blood cell, leading to platelet aggregation (Atsma et al., 2012; Kalra et al., 2017). In fact, hemoglobin levels have been reported to be positively associated with a variety of cardiovascular diseases, including atherosclerosis and ischemic stroke (Guo et al., 2019). Furthermore, low hemoglobin levels were significantly associated with mortality after AIS (Barlas et al., 2016; Kellert et al., 2011). As for erythrocytes, the changes of oxidative and proteolytic in red blood cell would lead to the cellular rheology, which further influenced inflammatory processes, thus affecting severity of ischemic stroke (Pretorius & Lipinski, 2013). Moreover, anemia and RBC levels had been proposed as predictive indicators in cardiovascular disease and mortality (Ye et al., 2020).

Lymphocyte and platelet played a leading role in inflammation and thrombosis in the pathogenesis of ischemic stroke. Lymphocyte, a well‐known prognostic element in heart disease, presented a capacity to give rise to acute physiological stress (Li et al., 2015). In many studies, as for subjects of lymphocyte, specific T cells (Macrez et al., 2011) and regulator T cells (Liesz et al., 2009) had a significant impact on eliminating the inflammatory response and regulating the protective function. Moreover, it has already been elucidated that systemic immunosuppression like lymphopenia could be induced by stroke (Yu et al., 2018). In the previous study, cortisol levels could predict short‐term outcomes and mortality after AIS (Tu et al., 2013). Lymphopenia could increase the level of pre‐stroke baseline cortisol and sympathetic tone (Kim et al., 2012), promoting the production of proinflammatory cytokines that may aggravate ischemic injury (Park et al., 2011) and thus influencing the clinical outcomes. Proved by recent studies, lymphocyte counts of patients died of acute cerebral infarction were lower than that of patients who survived (Gunes & Buyukgol, 2020). Platelet, with a physiological function of hemostasis, was also of crucial importance in acute ischemic stroke. It could not only release various inflammatory mediators, but also develop inflammatory pathways through interacting with cells nearby. In the first place, platelets accumulated where blood vessels were injured, inducing the production of thrombosis and thrombin, which resulted in the start of coagulation process. When platelets were activated, they were used to form atherosclerotic plaques. Via generating thrombosis and stimulating inflammatory reaction, plaques became more stable (Modjeski & Morrell, 2013). Nevertheless, it was excessive activation and aggregation of platelets that brought about thrombosis and then caused vascular occlusion, leading to ischemia in cardiovascular and cerebrovascular (Modjeski & Morrell, 2013). To be specific, atherothrombosis and inflammatory immune response were promoted by platelet‐lymphocyte aggregation through interaction between platelets and lymphocytes, which could worsen lesion progression (Wang et al., 2020; Xu et al., 2019).

To the best of our knowledge, it is our study that is the first to present the correlation between RCI at 24‐hr and 3‐month prognosis and mortality of AIS patients treated with r‐tPA thrombolysis. RCI is a new and composite biomarker. It has at least two advantages. One is that in the context of ischemic stroke, it acts a comprehensive indictor, combining prognosis of single hemoglobin, platelet, lymphocyte, and RBC. The second advantage is that being a ratio calculated under a credible formula, it is hence more stable and can secure more accurate prognosis results than a single blood parameter. Recent studies have reported that RCI reflects respiratory function. Patients with higher RCI level are prone to have lower FEV1/FVC and higher PCO_2_ values. And in arterial blood gas analysis, lower FEV1/FVC level is associated with an increased risk of stroke (Joo et al., 2015). Hypoxia, which may have adverse effects on cells in the ischemic penumbra, tends to cause secondary brain damage and then leads to a poor prognosis to stroke patients. But the process of arterial blood gas analysis is itself technically complex, during which arterial puncture can give rise to some possible complications, such as local hematoma and arterial thrombosis. And the analysis may require several attempts before success, which makes it not an ideal way for clinical prognosis. In contrast, RCI, an index based on complete blood count parameters, can be more easily obtained and utilized to evaluate short‐term mortality in stroke patients. At the same time, clinicians often need to assess the short‐term mortality rates of patients and reliable predictions lead to a better scheduling of supportive care and a more efficient allocation of resources. However, compared with other studies, there are several limitations in this study. The sample size of this study is correspondingly small. And due to the limitation of cross‐sectional studies, causal conclusions had not been established even when we corrected for potential confounders in the logistic regression. In previous studies, RCI is inversely proportional to the respiratory function and higher RCI level is associated with lower FEV1/FVC and higher PCO_2_. High PCO_2_ and hypoxia levels are assumed to adversely affect the recovery of the functional area of cerebral infarction after reperfusion. The specific mechanism needs to be further explored. In addition, there may be a selection bias in our study on account of our patients who are sorted from a single hospital.

## 5 CONCLUSION

In conclusion, our study demonstrated that RCI at 24 hr was significantly associated with 3‐month mortality in AIS patients with r‐tPA. Although the underlying mechanisms still need to be elucidated, RCI could nonetheless be a potential predictor of AIS patients.

## CONFLICT OF INTEREST

According to the author order above, Dr. Mei, Dr. Zhou, Dr. Gao, Dr. Huang, Dr. Yang, Dr. Zeng, Dr. Shen, Dr. Hu, Dr. Sun, Dr. Li, Dr. Huang, and Dr. Chen report no conflict of interest.

## 6 AUTHOR’ CONTRIBUTION

Conceptualization and design: XH and GC; Methodology: XH, GC, MQ, and XZ; Software: XZ and BG; Validation: XZ and BG; Formal Analysis: XZ, BG, HH, and CY; Investigation: XH, GC, and MQ; Resources: XH and GC; Data Curation: MQ, XZ, BG, HH, CY, TZ, JS, JH, FS, and SL; writing—original draft preparation: MQ, XZ, and BG; writing—review and editing: XH, GC, MQ, XZ, BG, HH, CY, TZ, JS, JH, FS, and SL; visualization: MQ, XZ, and BG; supervision: XH and GC; project administration: XH and GC.
